# Unexpected angular or rotational deformity after corrective osteotomy

**DOI:** 10.1186/1471-2474-15-175

**Published:** 2014-05-24

**Authors:** Seung Yeol Lee, Jiwon Jeong, Kyungho Lee, Chin Youb Chung, Kyoung Min Lee, Soon-Sun Kwon, Young Choi, Tae Gyun Kim, Jeong Ik Lee, Jehee Lee, Moon Seok Park

**Affiliations:** 1 Department of Orthopaedic Surgery, Seoul National University Bundang Hospital, Kyungki, Korea; 2 Department of Orthopaedic Surgery, Myongji hospital, Kyungki, Korea; 3 School of Computer Science and Engineering, Seoul National University, Seoul, Korea; 4 Biomedical Research Institute, Seoul National University Bundang Hospital, Kyungki, Korea; 5 Department of Orthopaedic Surgery, Konyang University Hospital, Daejon, Korea

**Keywords:** Codman’s paradox, Unexpected angulation, Femoral varization derotational osteotomy, Femoral derotation osteotomy

## Abstract

**Background:**

Codman’s paradox reveals a misunderstanding of geometry in orthopedic practice. Physicians often encounter situations that cannot be understood intuitively during orthopedic interventions such as corrective osteotomy. Occasionally, unexpected angular or rotational deformity occurs during surgery.

This study aimed to draw the attention of orthopedic surgeons toward the concepts of orientation and rotation and demonstrate the potential for unexpected deformity after orthopedic interventions. This study focused on three situations: shoulder arthrodesis, femoral varization derotational osteotomy, and femoral derotation osteotomy.

**Methods:**

First, a shoulder model was generated to calculate unexpected rotational deformity to demonstrate Codman’s paradox. Second, femoral varization derotational osteotomy was simulated using a cylinder model. Third, a reconstructed femoral model was used to calculate unexpected angular or rotational deformity during femoral derotation osteotomy.

**Results:**

Unexpected external rotation was found after forward elevation and abduction of the shoulder joint. In the varization and derotation model, closed-wedge osteotomy and additional derotation resulted in an unexpected extension and valgus deformity, namely, under-correction of coxa valga. After femoral derotational osteotomy, varization and extension of the distal fragment occurred, although the extension was negligible.

**Conclusions:**

Surgeons should be aware of unexpected angular deformity after surgical procedure involving bony areas. The degree of deformity differs depending on the context of the surgical procedure. However, this study reveals that notable deformities can be expected during orthopedic procedures such as femoral varization derotational osteotomy.

## Background

**Figure 1:**
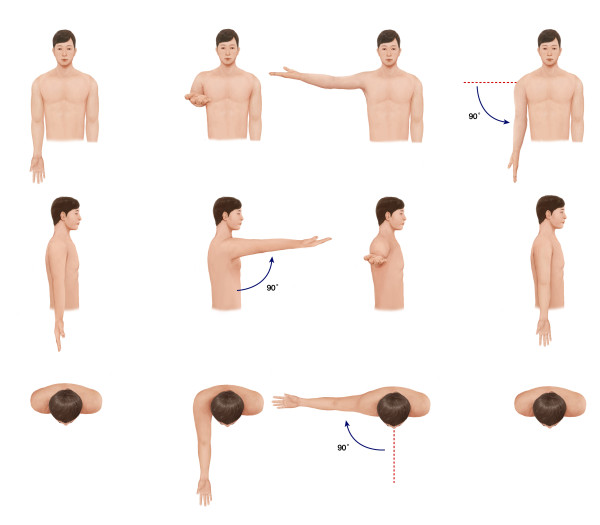
**Codman’s paradox.** A man places both arms along the body with the palms faced anteriorly. He elevates one arm 90 degrees forward without rotation. The palm faces superiorly. Following that, he abducts the same arm at 90 degrees horizontally. The palm still faces superiorly. Finally, the arm is adduced 90 degrees downward. Now, the direction of the palm is altered from the first direction.

Codman’s paradox is a specific pattern of motion at the shoulder joint [1]. In the anatomic position of the shoulder joint, the palm is positioned anteriorly. If the arm is forward elevated to 180 degrees and then descended without rotation on its long axis in the coronal plane to the side of the body, eventually, the palm will face posteriorly. However, if the arm is forward elevated to 360 degrees, the palm will face anteriorly. Many studies have attempted to solve this paradox mathematically [2-6], concluding that Codman’s paradox is not a paradox but the result of three closed-loop rotations of the long axis (Figure 1) [2,4]. Three closed-loop rotation involves three sequential long-axis rotations (forward flexion, abduction, and adduction) that form a closed loop. Codman’s paradox is caused by the swing of the arm about an axis that coincides with the long axis at the beginning of the three sequential arm rotations [2].

During arthrodesis of the shoulder for severe arthritis of the shoulder joint, a surgeon set the patient’s shoulder position to 30 degrees flexion, 20 degrees abduction, and 40 degrees internal rotation sequentially from the neutral position. However, after surgery, the surgeon found that internal rotation of the shoulder was less than 40 degrees because unexpected external rotation of the shoulder can occur during sequential movement of the arm.

A similar situation can occur during deformity correction in a long bone. For example, an orthopedic surgeon performed femoral varization derotational osteotomy for coxa valga and increased femoral anteversion. The surgeon planned to adduct the distal fragment 45 degrees with closed-wedge osteotomy and rotate the distal fragment 30 degrees externally [7]. After deformity correction, the surgeon found that the distal fragment was flexed or extended.

These issues occur frequently during surgical situations, such as a determination of the position of the arthrodesis and deformity correction for malunion with angular and rotation problems. Sometimes, these cases are beyond the surgeon’s intuition.

To understand this situation, the concepts of “orientation” and “rotation” with affine geometry may be helpful [8]. Affine geometry is defined as a combination of vectors and scholars. “Orientation” in affine geometry is defined as the vector, which represents where the object is headed. “Rotation” is defined as the scholar, which represents the amount of rotation of the object about a certain axis. In affine geometry, the two aforementioned examples denote a change of the orientation after a combination of two rotations about different axes. This change in orientation both puzzles orthopedic surgeons in their daily practice and affects the outcome of orthopedic procedures to correct deformities.

The purposes of this study were to present the concepts of orientation and rotation to orthopedic surgeons and demonstrate practical guidelines for deformity correction. To this end, we performed three simulations by means of a shoulder joint model using affine geometry, a model of femoral varization derotational osteotomy, and a three-dimensional (3D) reconstruction model of the femur for proximal femoral derotation osteotomy (FDO).

## Methods

This study was approved by the Institutional Review Board at Seoul National University Bundang Hospital (B-1202/145-108). Informed consent was waived owing to the retrospective nature of the study.

### Simulation 1

**Figure 2:**
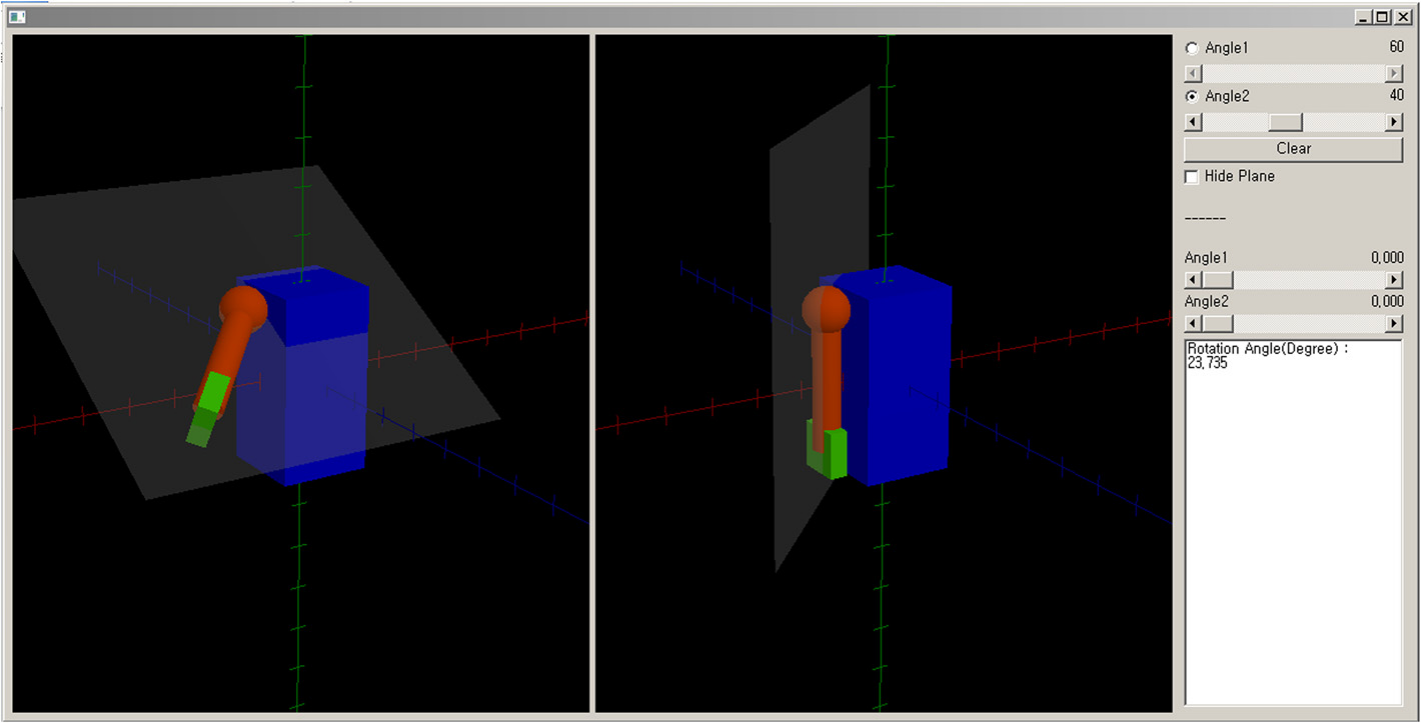
The shoulder motion model.

We simulated shoulder motion using a software tool developed by one of the authors using JAVA (Oracle Corporation, Redwood Shores, USA). With this model, forward flexion of the shoulder was initially accomplished. Then, abduction was performed along the plane of the forward flexed arm (Figure 2, left model). After forward flexion and abduction, the rotation of the shoulder relative to the initial position was calculated. The difference in the rotational angle between before and after the shoulder motion was calculated after the arm was lowered via the most direct route (along the gray plane on the right model of Figure 2) to the initial position without rotation of the shoulder joint (Figure 2, right model). We calculated the unexpected rotational angle after shoulder motion at every 10 degrees of forward flexion and abduction of the shoulder joint.

### Simulation 2

**Figure 3:**
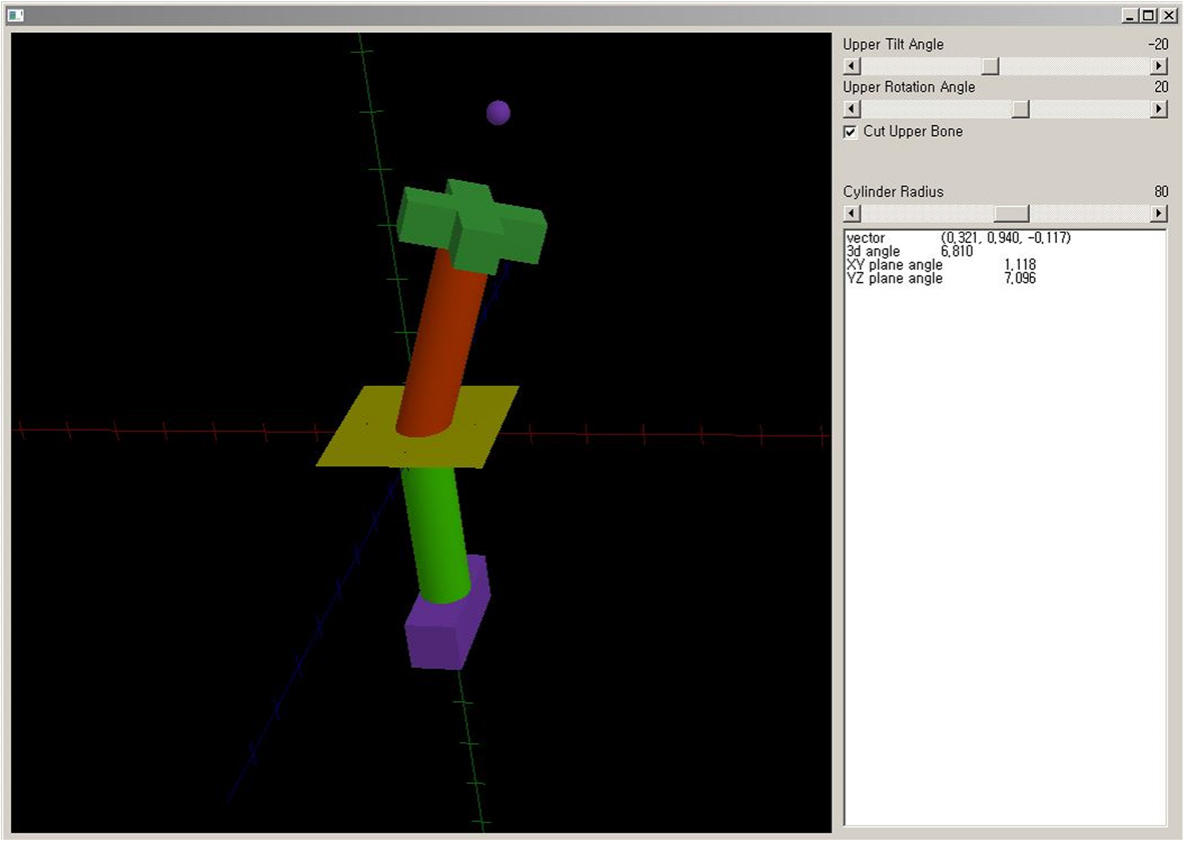
The varization and rotation model.

**Figure 4:**
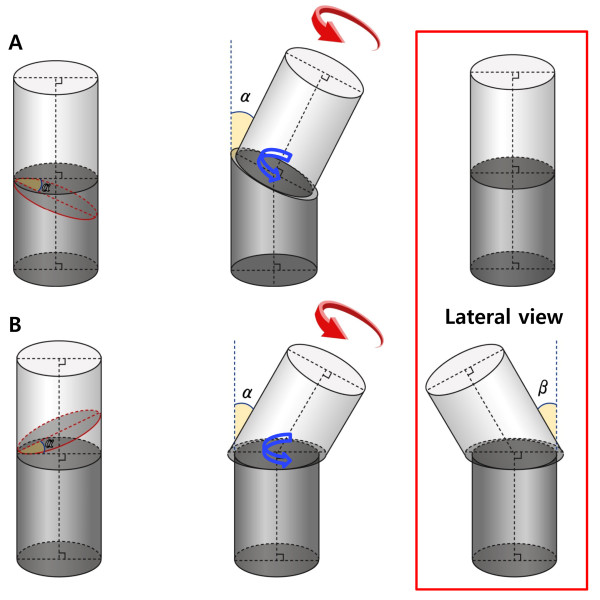
**The schematic model of the varization and rotation model.** In the varization and rotation model, closed-wedge osteotomy with a proximal cutting plane perpendicular to the axis of the long bone did not lead to unexpected angular deformity after rotation of the proximal fragment **(A)**. The actual rotation at the cutting plane (blue arrow) and rotation force to the proximal fragment (red arrow) occurred in the same direction in this case. Conversely, closed-wedge osteotomy, with a distal cutting plane perpendicular to the axis of the long bone, resulted in unexpected angular deformity after rotation of the proximal fragment. Note that the direction of actual rotation at the cutting plane (blue arrow) differs from that of the rotation force to the proximal fragment (red arrow) **(B)**. α, angle at the hinge point of the closed wedge; β, unexpected angular deformity on the sagittal plane.

We used a cylinder model to measure the unexpected angulation according to the direction of the cutting plane of the osteotomy site after closed-wedge osteotomy. Reorientations using a different cutting plane were simulated using a JAVA-based software tool developed by one of the authors (Figure 3). With this model, we simulated closed-wedge osteotomy in two situations according to the shape of the wedge. The first situation was to perform closed-wedge osteotomy with a proximal cutting plane perpendicular to the axis of the long bone. We assumed that the axes of the proximal and distal fragment were connected after reduction. The model was assumed to be a right limb. The distal fragment was assumed to be fixed, and the proximal fragment was then rotated on its axis internally. We calculated the 3D angle of the unexpected angular deformity of the long bone at every 10 degrees of rotation of the proximal fragment. The 3D angle was defined as the angle between the axes of the proximal fragment before and after rotation (Figure 4A). Contrary to the first situation, the second situation was to perform a closed-wedge osteotomy with a distal cutting plane perpendicular to the axis of the long bone. The proximal fragment was rotated on its axis internally on the assumption that the distal fragment was fixed. We calculated the 3D angle of the unexpected angular deformity of the long bone at every 10 degrees of rotation of the proximal fragment (Figure 4B). We hypothesized that the second situation is equivalent to femoral varization derotational osteotomy.

### Simulation 3

**Figure 5:**
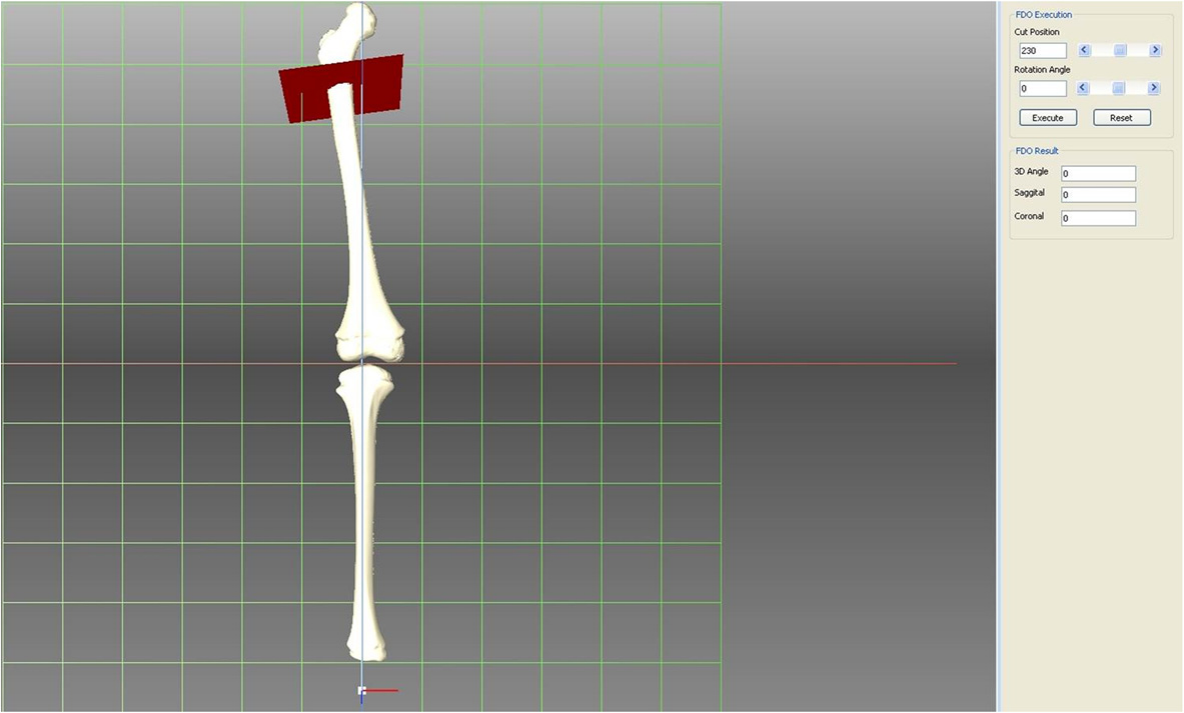
Three-dimensional reconstruction model of femoral derotation osteotomy.

We simulated proximal FDO, which is typically performed in patients with abnormalities of the femoral rotational profile. FDO is a procedure performed primarily to decrease femoral anteversion and thus improve the in-toeing gait in the transverse plane. Because FDO is usually performed with other procedures as a single-event multilevel surgery for patients with cerebral palsy at an age of 5–7 years [9], a computed tomography (CT) image of a 6-year-old boy was retrieved to create a 3D shape of the femur and tibia. 3D reconstruction of the right femur and tibia was performed with CT data using medical image processing software (Mimics, Materialise N.V., Belgium, version 14.11). After reconstruction of the femur and tibia, the mechanical axis of the lower extremity (mechanical axis), which begins at the center of the femoral head and continues to the center of the ankle, was drawn. We confirmed that the reconstructed CT displayed normal alignment of the lower extremity because the mechanical axis passed through the medial tibial spine. Femoral cutting and rotating were simulated using Visual Studio 2010 (Microsoft, Redmond, USA) (Figure 5). FDO was performed at the intertrochanteric level of the proximal femur. The anatomical axis, which is the bisecting line of the proximal femur on the coronal and sagittal planes, was calculated, and a cutting plane was then made perpendicular to the anatomical axis of the proximal femur. After cutting the proximal femur, external rotation about the axis of the proximal femur was performed on the distal fragment to the cutting site. The changes in the mechanical axis of the lower limb were calculated for every 10 degrees of rotation.

## Results

In the shoulder model, unexpected external rotation of the arm occurred after two-plane motion. The rotation angle of the shoulder after motion increased as the forward flexion or abduction angle increased (Table 1).

**Table 1:** Unexpected external rotation after forward flexion and abduction of shoulder joint

**Flexion**	**Abduction**								
	**10°**	**20°**	**30°**	**40°**	**50°**	**60°**	**70°**	**80°**	**90°**
10°	0.9	1.8	2.7	3.6	4.7	5.8	7.0	8.4	10
20°	1.8	3.6	5.4	7.3	9.4	11.6	14.1	16.8	20
30°	2.7	5.4	8.2	11.1	14.2	17.6	21.3	25.3	30
40°	3.6	7.3	11.1	15.1	19.3	23.7	28.6	34.0	40
50°	4.7	9.4	14.2	19.3	24.5	30.1	36.2	42.7	50
60°	5.8	11.6	17.6	23.7	30.1	36.9	44.0	51.7	60
70°	7.0	14.1	21.3	28.6	36.2	44.0	52.2	60.9	70
80°	8.4	16.8	25.3	34.0	42.7	51.7	60.9	70.3	80
90°	10	20	30	40	50	60	70	80	90

In the varization and derotation model, closed-wedge osteotomy with a proximal cutting plane perpendicular to the axis of the long bone did not lead to unexpected angular deformity after rotating the proximal fragment. In this situation, the angle of inclination of the cutting plane of the proximal fragment was 0 degrees. Conversely, closed-wedge osteotomy with a distal cutting plane perpendicular to the axis of the long bone resulted in unexpected angular deformity after rotating the proximal fragment. As the proximal fragment was rotated internally, extension on the sagittal plane and valgus on the coronal plane occurred. The valgus on the coronal plane and the 3D angle increased as the internal rotation angle increased, and extension on the sagittal plane increased as the internal rotation angle increased at an internal rotation of less than 60 degrees. As the angle at the hinge point of the closed wedge (angle α) increased, unexpected angular deformity to the axis of the distal fragment also increased (Table 2).

**Table 2:** Unexpected angular deformity after varization and derotation of the model according to inclination of the cutting plane

**Varization (angle α)**		**Derotation**					
		**10°**	**20°**	**30°**	**40°**	**50°**	**60°**
5°	3D angle	0.9	1.7	2.6	3.4	4.2	5.0
	Valgus	0.1	0.3	0.7	1.2	1.8	2.5
	Extension	0.9	1.7	2.5	3.2	3.8	4.3
10°	3D angle	1.7	3.5	5.2	6.8	8.4	10.0
	Valgus	0.1	0.6	1.3	2.3	3.5	5.0
	Extension	1.8	3.5	5.0	6.5	7.7	8.7
15°	3D angle	2.6	5.2	7.7	10.2	12.6	14.9
	Valgus	0.2	0.9	1.9	3.4	5.2	7.4
	Extension	2.7	5.2	7.6	9.8	11.6	13.1
20°	3D angle	3.4	6.8	10.2	13.4	16.6	19.7
	Valgus	0.3	1.1	2.5	4.4	6.8	9.7
	Extension	3.6	7.1	10.3	13.2	15.6	17.5
25°	3D angle	4.2	8.4	12.6	16.6	20.6	24.4
	Valgus	0.3	1.3	3.0	5.3	8.3	11.9
	Extension	4.6	9.1	13.1	16.7	19.7	22.0
30°	3D angle	5.0	10.0	14.9	19.7	24.4	29.0
	Valgus	0.4	1.5	3.4	6.1	9.6	13.9
	Extension	5.7	11.2	16.1	20.4	23.9	26.6
35°	3D angle	5.7	11.4	17.1	22.6	28.1	33.3
	Valgus	0.4	1.7	3.8	6.8	10.8	15.7
	Extension	6.9	13.5	19.3	24.2	28.2	31.2
40°	3D angle	6.4	12.8	19.2	25.4	31.5	37.5
	Valgus	0.4	1.7	4.0	7.3	11.7	17.2
	Extension	8.3	16.0	22.8	28.3	32.7	36.0
45°	3D angle	7.1	14.1	21.1	28.0	34.8	41.4
	Valgus	0.4	1.8	4.1	7.5	12.3	18.4
	Extension	9.9	18.9	26.6	32.7	37.5	40.9
50°	3D angle	7.7	15.3	22.9	30.4	37.8	45.0
	Valgus	0.4	1.8	4.1	7.6	12.5	19.2
	Extension	11.7	22.2	30.8	37.5	42.4	45.9
55°	3D angle	8.2	16.4	24.5	32.5	40.5	48.4
	Valgus	0.4	1.7	4.0	7.4	12.4	19.5
	Extension	13.9	26.0	35.5	42.6	47.6	51.0
60°	3D angle	8.7	17.3	25.9	34.5	42.9	51.3
	Valgus	0.4	1.6	3.7	7.0	11.9	19.1
	Extension	16.7	30.6	40.9	48.1	53.0	56.3

3D angle: angle between the axes of the proximal fragment before and after derotation.

In a femoral 3D reconstruction model of the right leg, the femoral neck shaft angle was 128.8 degrees, and the degree of femoral anteversion was 18.8 degrees. After FDO at the intertrochanteric area, unexpected varus on the coronal plane and extension on the sagittal plane were observed (Table 3). The angulation on the coronal plane was more affected than that on the sagittal plane. The 3D angle of the deviated mechanical axis was greater than 5 degrees when the derotation angle was larger than 33 degrees.

**Table 3:** Unexpected angulation of a mechanical axis of the lower extremity after femoral derotation osteotomy

**Osteotomy site**		**External rotation of distal fragment**					
		**10°**	**20°**	**30°**	**40°**	**50°**	**60°**
Intertrochanter	3D angle	1.6	3.1	4.7	6.2	7.6	7.6
	Varus	1.4	2.8	4.4	6.0	7.5	7.5
	Extension	0.8	1.3	1.6	1.6	1.3	1.3

3D angle: angle between the mechanical axes before and after derotation.

## Discussion

Occasionally, surgeons observe unexpected angular deformities after deformity correction procedures. In this study, a combination of forward elevation and abduction of the shoulder joint resulted in unexpected external rotation. Closed-wedge osteotomy and additional derotation can cause unexpected valgus deformity, name under-correction of coxa valga. In addition, because of the inequality of the mechanical and anatomic axes, varus angulation can be expected when performing FDO.

A limitation of this study should be addressed before discussing these findings in detail. We performed three computer simulations to understand rotation and orientation under several surgical conditions. These are theoretical situations, and practical application requires further study because of the diversity observed in individual cases.

Rotation means circular movement. Orientation is defined as the state of being oriented. Geometric computing pertains to the method of handling geographic entities such as vectors and points. A vector is a quantity that has both magnitude and direction, and the magnitude and angle between vectors can be calculated. Affine geometry is an extended concept of vector geometry. Contrary to vector space, which does not include points, affine space includes vectors, points, and related operations. To explain human motion in 3D space, an application of affine geometry is required.

Several studies have solved Codman’s paradox mathematically [2-6]. However, surgeons often cannot understand this area easily because of the complex terminology. Furthermore, these studies focused only on orthogonal arm rotations around the shoulder to calculate the motion mathematically. In the present study, we simulated this situation at various angles of the shoulder and found that two-plane motion of the shoulder resulted in external rotation of the shoulder. Surgeons should pay attention to unexpected rotation of the shoulder joint during shoulder positioning surgeries such as arthrodesis.

Femoral varization derotational osteotomy is indicated for femoral anteversion and coxa valga [10,11]. This surgery allows varus angulation of the femoral neck to ensure stability of the hip with internal rotation of the femur proximal to the osteotomy and external rotation distal to the osteotomy [10]. In our simulation, as the proximal fragment was rotated internally, extension occurred on the sagittal plane and valgus occurred on the coronal plane. For example, 7.3 degrees of valgization and 28.3 degrees of extension occurred after varization derotation osteotomy with 40 degrees of varization and internal rotation. This indicates under-correction during the coxa valga procedure. The extension angle after varization and derotation was larger than the valgus deformity angle. Surgeons should be aware of this issue.

The mechanical axis of the lower extremity should be considered when deformity correction is performed in the femur. The mechanical axis of the lower limb extends from the center of the femoral head to the center of the ankle joint and passes near or through the center of the knee. The mechanical axis of the lower extremity is at 3 degrees of valgus from the vertical axis of the body. The anatomical axis of the femur is at 6 degrees of valgus from the mechanical axis of the lower limb and 9 degrees of valgus from the true vertical axis of the body [7]. A previous study reported that foot rotation can affect the mechanical axis of the lower extremities [12]. Rotation of the bone through the right-angled cutting plane to the anatomical axis of the femur can affect the alignment of the lower leg. In addition, femoral anteversion and femoral bowing also can affect the alignment of the lower leg. In general, osteotomy during FDO is performed on a line perpendicular to the long axis of the femur [13]. The mechanical axis of the extremity can be affected by FDO, and further influences can be added if valgization or varization of the femur occurs. In practice, further study regarding the radiologic and functional results, such as gait analyses after surgery, will be needed.

## Conclusions

Surgeons should be aware of unexpected angular deformity after surgical procedures involving bony areas. The degree of deformity differs depending on the context of the surgical procedure. However, this study revealed that notable deformities can be expected during orthopedic procedures such as femoral varization derotational osteotomy.

## Abbreviations

FDO: Femoral derotational osteotomy; 3D: 3-dimentional; CT: Computed tomography.

## Competing interests

No potential conflict interest relevant to this article was reported.

## Authors’ contributions

All authors on this manuscript (SYL, JJ, KL, CYC, KML, SSK, YC, TGK, JIL, JL, and MSP) made significant contributions to the study design. SYL and MSP were involved in acquisition of data. JJ, KL, and JL developed the simulation software. All authors were involved in the analysis and interpretation of data, as well as drafting the manuscript. All authors gave final approval of the version to be published. All authors read and approved the final manuscript.

## Pre-publication history

The pre-publication history for this paper can be accessed here:

http://www.biomedcentral.com/1471-2474/15/175/prepub
